# Non-Alcohol Hand Sanitiser Gels with Mandelic Acid and Essential Oils

**DOI:** 10.3390/ijms24043855

**Published:** 2023-02-14

**Authors:** Pavlína Egner, Jana Pavlačková, Jana Sedlaříková, Pavel Pleva, Pavel Mokrejš, Magda Janalíková

**Affiliations:** 1 Department of Fat, Surfactant and Cosmetics Technology, Faculty of Technology, Tomas Bata University in Zlín, Vavrečkova 275, 760 01 Zlín, Czech Republic; 2 Department of Environmental Protection Engineering, Faculty of Technology, Tomas Bata University in Zlín, Vavrečkova 275, 760 01 Zlín, Czech Republic; 3 Department of Polymer Engineering, Faculty of Technology, Tomas Bata University in Zlín, Vavrečkova 275, 760 01 Zlín, Czech Republic

**Keywords:** antimicrobial activity, essential oils, mandelic acid, gels, stability testing

## Abstract

Antimicrobial hand gels have become extremely popular in recent years due to the COVID-19 pandemic. Frequent use of hand sanitising gel can lead to dryness and irritation of the skin. This work focuses on the preparation of antimicrobial acrylic acid (Carbomer)-based gels enhanced by non-traditional compounds—mandelic acid and essential oils—as a substitute for irritating ethanol. Physicochemical properties (pH and viscosity), stability and sensory attributes of the prepared gels were investigated. Antimicrobial activity against representative Gram-positive and Gram-negative bacteria and yeasts was determined. The prepared gels with mandelic acid and essential oil (cinnamon, clove, lemon, and thyme) proved to have antimicrobial activity and even better organoleptic properties than commercial ethanol-based antimicrobial gel. Further, results confirmed that the addition of mandelic acid had a desirable effect on gel properties (antimicrobial, consistency, stability). It has been shown that the essential oil/mandelic acid combination can be a dermatologically beneficial hand sanitiser compared to commercial products. Thus, the produced gels can be used as a natural alternative to alcohol-based daily hand hygiene sanitisers.

## 1. Introduction

The skin of a healthy person contains resident bacteria. Transient microbiota from the environment can cause an infection or even a severe health disease [1]. Hand hygiene, including hand washing and hand disinfection, is considered an important and effective measure against the transmission of microorganisms. During recent pandemics, hygienic hand disinfectants have received widespread attention, especially in gel form [2]. The gel is considered to be a cosmetic vehicle of many forms, which can serve as a carrier of active substances transported to the target site. The consistency and texture of the gel are modifiable and may be tailored to the specific application [3,4]. The gel easily turns into a liquid and is quickly absorbed into the skin, which reduces the possibility of run-off. This is accompanied by lower consumption and economic benefits [1].

Recently, the global market has provided a wide range of gel products with antimicrobial properties, especially alcohol-based gels. In international standards and guidelines, alcohols are recognised to be the most effective and efficient antimicrobials with a vast spectrum of antimicrobial activity [5]. The recommended ethanol/isopropanol/n-propanol concentration is usually in the range of 60–95% *v*/*v*, which is suggested to control the occurrence of dangerous microorganisms on the hands. The disinfecting effect of alcohols increases with molecular weight and chain length [6]. Their mechanism of antimicrobial action is based on the ability to denature proteins. All vegetative forms of Gram-positive and Gram-negative bacteria are very sensitive to alcohols except staphylococci. Furthermore, alcohols are not sporicidal, and bacterial spores can survive in alcohol solutions for several years [7]. Alcohol-based disinfectants are safe, fast acting, and not associated with resistance. On the other hand, alcohols can be irritating in contact with sensitive or damaged skin and prolonged healing can lead to skin disease or infection. They dissolve lipid molecules on the skin, leading to dehydration and forming small cracks [8]. All hand hygiene products should be active against nosocomial pathogens. Their efficacy is being tested according to European Standard EN 1500, where the tested preparation should not be significantly less efficient than the reference product [9].

Recently, attention has focused more on natural antimicrobials (especially essential oils—EOs). Their antimicrobial activity depends on their concentration and exposure time [10] Many studies document the medicinal properties of essential oils, such as anti-inflammatory, healing or antimicrobial activities, but may also be responsible for negative qualities such as photosensitivity and toxicity [11,12,13]. A strong correlation between chemical composition and antimicrobial activity has been proved [14]. It is known that the chemical composition of EOs is significantly affected by their origin, locality, period of harvest and processing conditions. The antimicrobial properties of essential oils are mainly caused by their components, isoprenes, such as monoterpenes, sesquiterpenes and related alcohols, hydrocarbons and phenols [15]. Limonene [14], cinnamaldehyde [13], eugenol [13], and thymol [16] are the major components of lemon, cinnamon, clove and thyme EO, respectively. The principle of their antibacterial action consists in the activity of included phenolic compounds breaking the microbial cell membranes, which finally results in leaking, disorder and death [17].

The antibacterial activity of EOs is based on their chemical composition. However, each constituent can play a key role not only alone, but may also interact with another one or more major or minor compounds. The greater antimicrobial effect may be due to an additive effect, which is equal to the sum of individual constituents or synergy, which means an effect bigger than the sum of individual constituents [18]. The synergic activity of the combination of citral, vanillin, thymol, eugenol or carvacrol was found against *Zygosaccharomyces bailii.* On the other hand, antagonism was observed for some substances, mainly the effect between p-cymen, tymol and carvacrol (Lippia chevalieri oil) [15]. In recent years, there has been increasing evidence that not only the ratio in which main compounds were presented in essential oils but also the interactions between them and minor constituents are both factors responsible for the inherent antimicrobial activity of EOs [18].

Mandelic acid is another substance that has been extensively studied for its antimicrobial properties. This alpha-hydroxy acid is a nontoxic substance with a long history of use in medicine as an antibacterial, particularly in treating urinary tract infections. It acts as an antiseptic against various microbial pathogens such as *Staphylococcus aureus*, *E. coli*, *Proteus* sp., *Pseudomonas* sp. [19,20,21]. Nowadays, dermatologists suggest mandelic acid as a useful agent for a wide variety of skin problems, from acne to wrinkles. Mandelic acid has been studied extensively for its possible uses in treating common skin problems such as photoaging, irregular pigmentation and acne [19].

The aim of this study was to prepare an antimicrobial hand gel based on essential oils and mandelic acid as an alternative to alcohol. The antimicrobial activity and sensory properties of the prepared products were investigated and compared with commercial hand gels. Long-term thermal stability was assessed to evaluate the potential of prepared gels for practical applications.

## 2. Results

The antimicrobial hand gels based on essential oil and mandelic acid were prepared and the following measurements were performed—antimicrobial activity, stability testing and sensory analysis.

### 2.1. Antimicrobial Activity In Vitro

The antimicrobial activity was tested by the disk diffusion method either on paper disks in the case of mandelic acid and essential oils or in wells in agar in the case of prepared gels.

#### 2.1.1. Mandelic Acid and Essential Oils

The disk diffusion method was used to determine the antimicrobial activity of substances that were subsequently used to prepare gels, namely mandelic acid (5, 10 and 15% solutions) and essential oil (EO—cinnamon CEO, clove CLEO, lemon LEO and thyme TEO). The results summarised in Table 1 show that all tested microorganisms were sensitive to all tested compounds except for lemon essential oil, which did not show any inhibitory activity against Gram-negative bacteria (*E. coli* and *P. aeruginosa*). On the other hand, the highest antimicrobial activity was observed by TEO, followed by CLEO and CEO. The antimicrobial effect of MA itself depended on its concentration. Already 10% of MA showed significant antimicrobial activity against all tested microorganisms.

#### 2.1.2. Gels with EO or/and MA

Antimicrobial activity of GEOs, GMAs, and GEO/MAs was assessed against bacteria and yeasts. The results are shown in Table 2. GMAs did not show an inhibitory effect against yeasts and bacteria, except against Gram-positive *S. aureus* and GMA (15%) was also active against *P. aeruginosa*. The gels containing only EO (CEO, CLEO, and TEO) demonstrated an antimicrobial effect, whereas GLEO showed no antimicrobial activity. On the other hand, the addition of MA enhanced the antimicrobial activity of GEOs, especially in the samples containing LEO/MA (15% solution). Therefore, the gels containing EO and 15% MA solution were used for further experiments of antimicrobial activity in vivo (see Section 2.2).

Gels containing Et (45 and 60%) were also prepared and tested to compare their antimicrobial effect with alcohol-free samples. GEt (45%) showed very low antifungal and antibacterial activity, whereas a higher Et concentration (60%) caused an increase in antimicrobial effect. GEO samples, except GLEO, exhibited comparable activity against *P. aeruginosa* and *S. aureus* and even better antimicrobial activity against *M. luteus* and yeasts than GEt 60%. The addition of MA significantly enhanced the antimicrobial activity, as was proved even by GCEO/MA samples against *E. coli*.

### 2.2. Antimicrobial Activity In Vivo

**Figure 1:**
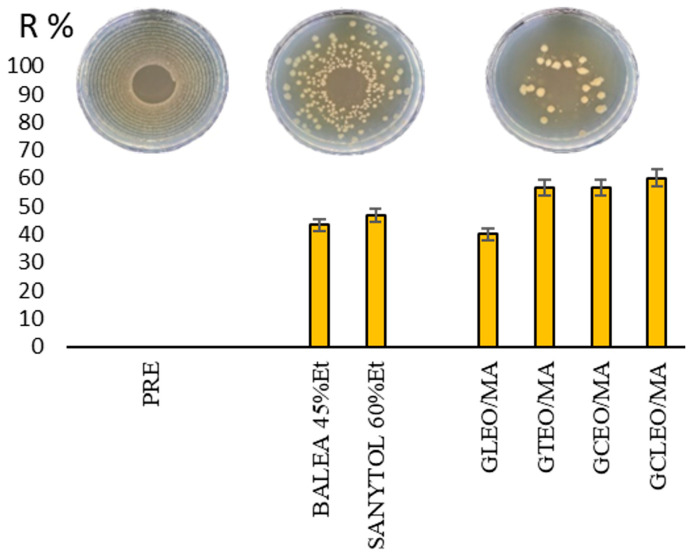
The percent reduction in microbial counts after application of the tested gels (BALEA 45%Et, SANYTOL 60%E, GLEO/MA, GTEO/MA, GCEO/MA, and GCLEO/MA) under practical conditions. PRE illustrates the artificially contaminated microbiota of hands without hand gel use. Petri dishes illustrate the antibacterial effect of GCEO/MA gel during in vivo testing.

The antimicrobial efficacy tests of prepared gels (GEO/MAs) in vivo were intended to simulate the practical conditions of hand sanitiser usage. The percent reduction in microorganisms (Figure 1) presents the efficacy of the tested gels after antimicrobial hand gel application according to EN 1500.

The results reveal that the highest antimicrobial activity was observed with GCLEO/MA, followed by GCEO/MA and GTEO/MA. In comparison, GLEO/MA showed the lowest efficacy, nevertheless, similar to ethanol-based tested commercial samples. The hand microbiome is highly variable because it is affected by different surrounding factors. This study confirmed that prepared GEO/MAs reduced microbial counts of the artificially contaminated hands (except GLEO/MA) significantly better than ethanol-based commercial products.

### 2.3. Stability Testing

#### 2.3.1. Effect of Temperature and Storage Time on pH and Viscosity

**Figure 2:**
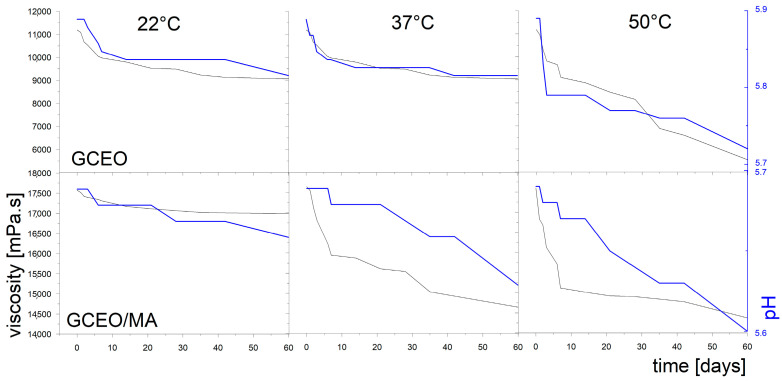
The viscosity and pH measured for GCEO and GCEO/MA gels during 60 days.

The pH was measured with time up to 60 days at three different temperatures (22 ± 1 °C, 37 ± 1 °C and 50 ± 1 °C). It was found that the temperature and storage time had no significant effect on the pH of all GEOs (Figure 2 and Figure S1). The mandelic acid in GEO/MAs caused even more negligible decrease in pH during the time compared to GEOs. The results generally showed a slight drop in pH, regardless of temperature and storage time.

As can be seen in Figure 2 and Figure S1, the most significant reduction in viscosity was observed at the highest tested temperature, 50 °C, in all the samples due to the destruction of the gel structure network. At lower temperatures, the viscosity reduction was slower and more gradual, except in the GLEO gel, when viscosity dropped more than 50%.

A significant decrease in the viscosity of GEOs occurred at 22 ± 1 °C within the first 10 days, after which the viscosity stabilised at approximately 6000 mPa.s. For gels containing TEO, CLEO and CEO, a slower and more gradual viscosity reduction was observed.

In the case of GEO/MA samples, no significant effect of MA addition on the viscosity was observed in GLEO samples during the entire testing period. On the other hand, a stabilising effect of mandelic acid on the gel structure can be confirmed in the samples with CLEO and CEO at almost all tested temperatures. Surprisingly, the TEO gel showed the opposite trend, with the most substantial drop (from approximately 17,000 to 2000 mPa.s) at 50 °C.

Comparison of the viscosity of the two series of samples, GEO and GEO/MA, showed that increasing temperature significantly reduced the viscosity of gels containing only EO. On the other hand, the addition of MA enhanced the gel stability and kept the viscosity high except GLEO/MA.

#### 2.3.2. Effect of Temperature and Storage Time on Antimicrobial Efficacy and Organoleptic Properties

**Figure 3:**
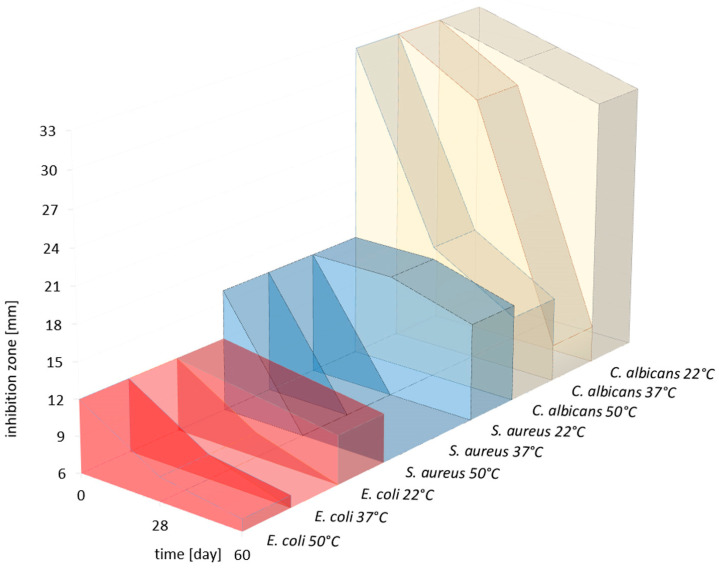
Antimicrobial effect against Gram-negative bacteria (*E. coli*), Gram-positive bacteria (*S. aureus*) and yeasts (*C. albicans*) of GCEO/MA at three temperatures (22 ± 1 °C, 37 ± 1 °C, and 50 ± 1 °C) during 60 days storage.

The antimicrobial activity of produced gels with mandelic acid (GEO/MA) against *E. coli*, *S. aureus*, and *C. albicans* was determined by the well diffusion method. Tests were performed immediately after the gel production (0 days) and after 28 and 60 days of storage at three temperatures (22 ± 1 °C, 37 ± 1 °C, and 50 ± 1 °C). The results for GCLEO/MA, GLEO/MA, and GTEO/MA are presented in Figure 3 (GCEO/MA) and Table S1 (Supplementary).

All GEO/MAs stored at 22 ± 1 °C exhibited promising antimicrobial activity against all tested microorganisms even after 60 days of storage in comparison with higher tested temperatures. With increasing temperature, the antimicrobial efficacy decreased, especially against Gram-positive *S. aureus.* The decrease in effectivity can probably be explained by EO oxidation at a higher temperature. It was proved that the most significant effect GCEO/MA was against yeasts and Gram-positive bacteria. The comparable activity of GCEO/MA and GTEO/MA was noticed against Gram-negative bacteria. The lowest activity of all tested gels had GLEO/MA.

**Figure 4:**
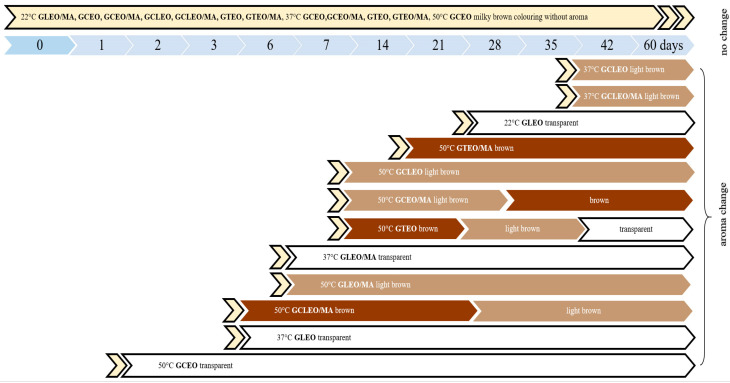
Physical characteristics of the prepared gels (odour and colour).

The visual and odour properties (colour and smell) of the produced gels (GEO and GEO/MA—15% solution) at defined time intervals were monitored (Figure 4). These properties should maintain at an acceptable level throughout the time. All produced gels were milky cloudy with a corresponding typical smell of the respective EO after their preparation. This coloration was caused by the addition of EO. The smell of the produced gel changed along with the colour change over time and with increasing storage temperature.

### 2.4. Sensory Analysis

**Figure 5:**
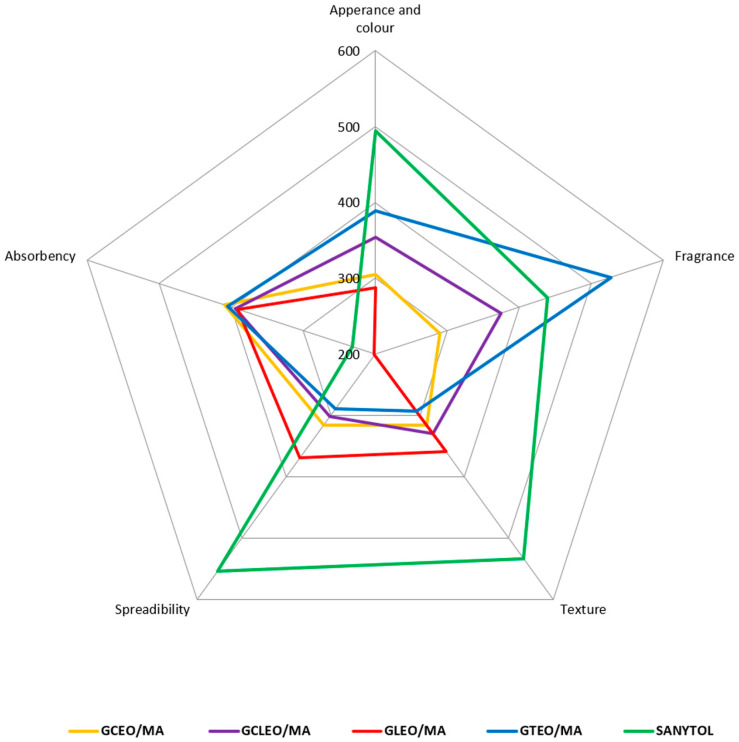
Organoleptic evaluation of prepared gels (rank sums for each property—Kruskal–Wallis test).

The organoleptic properties of the set of gel samples were assessed immediately after preparation with scales and subsequently evaluated with the Kruskal–Wallis test, which uses rank sums in the calculation algorithm (Table 3). The ranking sums correspond to the interpretation of the assessed properties supplemented by testing the differences between the gel samples at the 5% significance level. Sensory evaluation profiles were created from the five obtained characteristics, see Figure 5.

The appearance and colour were most often assessed as very good, corresponding to products of the given type, transparent with a low presence of bubbles. Based on the sum of the ranks, GLEO/MA sample was evaluated as the best, whereas ethanol-based commercial gel SANYTOL received the highest score. A statistically significant difference in this characteristic was not found. As regards the fragrance parameter, GLEO/MA was again the best-rated sample, while GTEO/MA was given the highest number of points. The calculation revealed conclusive statistical differences, specifically between the samples GCEO/MA—GTEO/MA, GLEO/MA—SANYTOL and GLEO/MA—GTEO/MA.

GTEO/MA with the typical gel texture received the lowest sum, while commercial SANYTOL gel was graded with the highest. There was a statistically significant difference between the SANYTOL—GTEO/MA gel samples. The results of spreadability are consistent with these texture founding, and GTEO/MA was identified as the best spreadable. From the set of samples, the commercial ethanol-based gel was marked with the least satisfactory spreadability. There was a statistically significant difference in this monitored property between the GCEO/MA—SANYTOL, GCLEO/MA—SANYTOL and GTEO/MA—SANYTOL samples. Absorbency was the last monitored characteristic using scales. The commercial SANYTOL gel showed very good and fast absorbency. On the other hand, GCEO/MA received the highest score, with poor absorbency resulting in a sticky film on the skin. However, no statistically significant difference in absorbency was found among the samples.

The evaluation of the sequential preference test proved that the evaluated samples did not differ in preferences. Based on the sum of the rankings, see Table 3, the assessors preferred GLEO/MA, followed by GCEO/MA, GCLEO/MA, gel with TEO/MA, and the commercial gel SANYTOL was marked last in the ranking.

## 3. Discussion

This work aimed to prepare hand sanitiser with alternative non-alcohol antimicrobial agents to avoid the adverse skin effect of ethanol. As Houben et al. [22] examined, it was found that a higher concentration of ethanol resulted in increased skin scaling during tests with six alcohol-based antimicrobial gels.

At the initial stage, the antimicrobial properties of the used EOs, MA and their combination were tested. The results showed that the antimicrobial activity of essential oils is mainly affected by their specific composition, functional groups present in active components, and their synergistic interactions [23]. Therefore, their antimicrobial efficacy in produced gels can be different as well. For example, the antimicrobial effects of CEO can be attributed to its main component, cinnamaldehyde, which can disrupt the membranes of bacteria, leading to cell lysis [24]. Goñi et al. also investigated the antibacterial activity of CEO in combination with CLEO against *Escherichia coli*, *Yersinia enterocolitica*, *Pseudomonas aeruginosa*, *Salmonella choleraesuis*, *Staphylococcus aureus*, *Listeria monocytogenes*, *Enterococcus* and *Bacillus* [25]. The main components responsible for the antimicrobial activity of TEO include carvacrol, thymol, p-cymene, ɣ-terpinene [16]. Cinnamaldehyde, eugenol, limonene and thymol, as main components of essential oils used in this study, have been shown to have also antiviral activities [26]. Other characteristics, such as solubility in water and ability of hydrogen bonding play important role in final antimicrobial activity, too. It has been already reported in the literature that although thymol, carvacrol and eugenol exhibit strong antimicrobial effects, these can be eliminated by high water solubility. This can be the reason of lower activity obtained in CLEO samples [27]. Thymol and carvacrol, as two components with highly similar structure, have been proved to have additive interaction [28]. It was found that the combination of linalool and eugenol exhibited a synergistic effect [18].

The antimicrobial activity of EOs was also confirmed in other works [29,30]. It is generally known that Gram-positive bacteria are more sensitive to the action of EOs than Gram-negative bacteria, mainly because of the cell wall structure [31,32]. According to Nazzaro et al. [32], the antibacterial effect of EOs and their components are associated with their lipophilic nature, which allows them to accumulate in membranes and thus act on cell wall destruction. Some studies [33,34] reported that selected EO could replace conventional antimicrobials. The addition of MA enhanced the antimicrobial effects of prepared gels. Similarly, Stickler et al. proved the in vitro antimicrobial properties of MA and their derived compounds. The effectiveness of MA (1% *w*/*v*) and MA (0.5% *w*/*v*)/lactic acid (0.5% *v*/*v*) mixture in eliminating the biofilm forming organisms related to urinary tract infections was shown [35].

The hand microbiome was found to be a more temporal variable than in other body sites. It consists of bacteria, fungi, viruses, and protozoa. Bacteria from four phyla were observed—*Firmicutes*, *Actinobacteria*, *Proteobacteria*, and *Bacteroidetes*. The hand microbiome is affected by temporal and biogeographical dynamics, as well as intrinsic and extrinsic factors [36]. While *Staphylococcus aureus* is a common cause of skin infection, methicillin-resistant *S. aureus* (MRSA) represents a severe cause of morbidity and mortality in many hospitals worldwide. The hands of health care workers are the most common vehicle for transmitting pathogens, so hand hygiene is very important in preventing and controlling MRSA [37]. MA derived compounds were tested for their antimicrobial activity against a few Gram-positive and Gram-negative bacteria by the disk diffusion method. They demonstrated the antimicrobial effect of MA at concentrations of 20–160 mg/mL against MRSA [19]. Thus, mandelic acid could be an effective treatment for MRSA and other bacterial skin infections.

Evidence from the literature search shows that several antimicrobial gels have been recently investigated [38,39,40]. Liu et al. [39] described the fabrication of PEG hydrogels that incorporate an antimicrobial polycarbonate (polycarbonate containing quaternary ammonium groups), forming cationic PEG-APC (polyethyleneglycol-antimicrobial polycarbonate) hydrogels. These gels showed strong antimicrobial activity against *S. aureus*, *E. coli*, and *C. albicans*. In addition, the prepared hydrogel proved to maintain its activity over time even when challenged daily with *S. aureus* for 12 days.

The results from the present study revealed that the antimicrobial activity of GEO and GEO/MA samples was significantly affected by the temperature and total storage time. The findings correspond to the work of Turek et al. [41], which states that rising temperature alter the stability of EOs and their chemical composition during storage. Those changes can negatively influence the resulting antimicrobial efficacy.

The viscosity of the gels plays a crucial role in their functionality, as the high viscosity facilitates contact time. Hydrogel preparations can be more desirable than liquid forms, especially from the viewpoint of user comfort. They absorb faster, have a shorter drying time, and leave a pleasant feeling on the hands. In addition, they can reduce the rate of alcohol evaporation [42]. Regarding the organoleptic and rheological properties of gel formulations, they are generally influenced by the used thickeners, active substances, and their concentrations [43]. In topical formulations, they may be perceived differently by the end-user when applied to the skin, not only during but also after application. The sensory influence of the ingredients was described, for example, in the publication [44], which investigated skin protection hydrophilic gels based on polyacrylates. The acrylic derivates have proven as good thickeners creating hydrophilic films on the surface of the skin. Hydroxyethyl cellulose and Carbomer of synthetic nature in the studied gels were used as gelling agents in the study of Savary et al. [45], who confirmed the good absorption properties of prepared samples. Similarly, the gels prepared in this study exhibited optimal absorption properties when no residual film was observed after their application on the skin. On the other hand, the tested commercial gel was evaluated as poorly spreadable, which can be the result of its thin texture. This indicates that the developed antimicrobial formulations outperformed the commercial formulation, especially in this subjectively assessed parameter.

## 4. Materials and Methods

### 4.1. Materials, Chemicals and Microorganisms

Essential oils (EO) were all obtained from Nobilis Tilia (Czech Republic): cinnamon (CEO), clove (CLEO), lemon (LEO), and thyme (TEO). The chemical composition of EOs is shown in Table S2 (Supplementary). Data were obtained from Nobilis Tilia (Czech Republic). Mandelic acid (MA), 96% ethanol (Et), and NaOH were purchased from Sigma-Aldrich (MO, USA). All gels prepared in this study (GEOs—gels with EOs; GMAs—gels with mandelic acid; GEO/MAs—gels with combination of EO and MA; GEts—gels with ethanol) were based on Carbomer (Polygel CA; Mica&Harasta, Czech Republic). SANYTOL with 60% ethanol (Marca, Czech Republic) and BALEA with 45% ethanol (dm-drogerie markt, Germany) were used as commercial ethanol-based hand sanitiser gels.

Diffusion methods were used for four bacterial and two yeast strains. Bacterial strains *Escherichia coli* CCM 3954, *Staphylococcus aureus* CCM 3953, *Pseudomonas aeruginosa* CCM 3955, *Micrococcus luteus* CCM 734, and yeast strains *Candida albicans* CCM 8275, and *Candida parapsilosis* CCM 8276 were supplied by Czech Collection of Microorganisms (Brno, Czech Republic). Bacteria were cultivated on Nutrient agar or Mueller Hinton agar; yeasts were grown on Sabouraud agar (Hi-Media Laboratories Ltd., Mumbai, India).

### 4.2. Preparation of Gels

Four types of gels were prepared: GEt (with ethanol), GEO (with essential oils), GMA (with mandelic acid), and GEO/MA (with essential oils and mandelic acid). The formulations of prepared gels are shown in Table 4.

The gel base was Carbomer (0.5 wt%), which was weighed into a 250 mL beaker, and demineralised water was supplemented (100 g). The swollen Carbomer gel base and ingredients (Et or EO) were thoroughly mixed for 10 min on a stirrer (MM4 LAVAT, CZ). Subsequently, GEt, and GEO gels were adjusted to pH 6 with 1 M NaOH solution.

For the preparation of gels enriched with mandelic acid (GMA), MA solutions of different concentrations (5, 10, 15%) were initially prepared. Then, the pH was adjusted to approx. 3.5 using 1 M NaOH solution. This acidified MA solution was added in the appropriate amount (5.0 wt%) to the swollen Carbomer gel base. In the case of GEO/MA gels, the addition of acidified MA solution was followed by the incorporation of the corresponding essential oil (0.5 wt%). The mixture was stirred vigorously for 10 min (MM4 LAVAT, CZ), and the pH of this solution was adjusted to pH 5.6–6.0 with 1 M NaOH solution.

### 4.3. Viscosity Measurement

The viscosity of the produced gels was measured at laboratory temperature (22 ± 1 °C) on a rotational viscometer MYR V2-L (Viscotech Hispania, SL, Spain) using the Brookfield method and evaluated using ViscosoftPlus software according to ISO 2555 [46]. All experiments were measured in triplicate.

### 4.4. pH Measurement

A pH meter (pH Spear Waterproof, USA) with a measurement accuracy of ± 0.1 pH was used to measure the pH of the produced gels. All measurements were taken at least three times for each value.

### 4.5. Antimicrobial Activity In Vitro

As the first step, the antibacterial activity of essential oils, mandelic acid and ethanol (control) was tested by the standard agar disk diffusion technique [47]. The sterile 6 mm disks (Whatman, Maidstone, UK) were impregnated with 10 µL active substance. They were placed on Mueller Hinton agar plates (Himedia Laboratories Pvt. Ltd., Mumbai, India) inoculated with 0.5 McF turbid bacterial suspension (*M. luteus*, *S. aureus*, *E. coli* and *P. aeruginosa*) in sterile saline solution and on Sabouraud agar (Himedia Laboratories Pvt. Ltd., Mumbai, India) inoculated with 0.5 McF turbid yeast suspension (*Candida albicans*, *C. parapsilosis*). The plates were incubated for 24 h at 37 °C for bacteria and 5 days at 25 °C for yeasts. The inhibition zones were measured in diameter, including the paper disk. All experiments were repeated three times.

The antimicrobial activity of prepared gels was tested by the well diffusion method, as was described earlier [48]. At first, 1 mL of each microbial suspension (0.5 McF) was pipetted into separate sterile Petri dishes and poured with 20 mL molten Mueller–Hinton agar (bacteria) or Sabouraud agar (yeasts). After solidification, wells of 5 mm diameter were made in the center of each agar plate by sterile cork borer, and 50 µL of gel was placed into it. The diameters of inhibition zones were measured. All experiments were performed at least in triplicate.

### 4.6. Antimicrobial Activity In Vivo

The efficacy of hygienic handrubs (GEO/MA) was tested by measuring the number of viable bacteria remaining on the fingertips of 18 volunteers according to EN 1500 [9]. All accepted volunteers had healthy, intact skin and they provided informed consent. Ethical approval was sought, but this was not required as the method used was based upon published EN standards, as well as institutional review board approval was not required in case volunteers involved in sensory analysis. Nevertheless, the selection of volunteers and the testing procedure were in accordance with international ethical principles of biomedical research with human participants [49]. This standard is designed to evaluate the ability of routine hand hygiene products to eliminate transient pathogens. This procedure involves the placement of bacteria on the hands followed by exposure to the test gel sanitiser [50]. The nonpathogenic bacterial strain *Escherichia coli* CCM 3954 was used in this study. Participants were asked to wash and dry their hands. The following step included artificial contamination of hand fingertips in bacterial suspension without (pre-value A) and with (post-value B) the hygienic handrub. Four GEO/MA with cinnamon, clove, lemon and thyme essential oil were tested. Two commercial ethanol-based hand sanitiser gels were used as comparative controls (BALEA 45% ethanol, SANYTOL 60% ethanol). Colony counts were obtained and log reductions were calculated. The logarithmic reduction factor was then expressed as a percent reduction. Log reduction was calculated according to Equation (1) [51]:

(1) $$R\;[\%]=\frac{A-B}{A}\cdot 100$$

where

*A* is a Log number of viable microorganisms without treatment (pre-value);

*B* is a Log number of viable microorganisms with treatment (post-value).

### 4.7. Stability Testing

Based on the guidelines on the stability testing of cosmetic products [52], stability tests (viscosity, pH, colour and odour changes) were performed with all prepared gels at 22 ± 1 °C, 37 ± 1 °C and 50 ± 1 °C for 60 days (0, 1, 2, 3, 6, 7, 14, 21, 28, 35, 42 and 60 days). The pH (pH Spear Waterproof, USA), the viscosity (MYR V2-L, Viscotech Hispania, SL, Spain) [46], colour and odour changes were measured at the same intervals. Antimicrobial activity was determined on days 0, 28 and 60. Each experiment was performed in triplicate.

### 4.8. Sensory Analysis

Sensory analysis was performed by a panel of 12 sensory assessors with prior sensory evaluation experience (ages 21–26 years). They were familiarised with the process and purpose of sensory evaluation through questionnaires. The sensory evaluation and equipment of the sensory laboratory of Tomas Bata University, Faculty of Technology, met the defined conditions according to the international standards ISO 6658 [53] and ISO 8589 [54]. The laboratory temperature ranged from 20 to 22 °C, and the room was illuminated by artificial lighting. Sensory evaluation of gels was performed after signing informed consent before accepting participation in the study by all sensory assessors.

The sample formulations were assessed by scales and a ranking preference test. Properties such as appearance and colour were evaluated using an ordinal hedonic five-level scale (1—excellent, typical of the type of formulation, transparent, presence of bubbles, 2—very good, appearance still adequate, transparent, moderate presence of bubbles, 3—good, transparent or slightly cloudy, no bubbles, 4—less good, appearance different, slightly yellowish, cloudy, no bubbles, 5—unsatisfactory, non-transparent, markedly cloudy and discoloured, no bubbles); smell (1—excellent, pleasant smell, 2—very good, no disturbing odours, 3—good, slightly disturbed by odours, 4—less good, much disturbed by odours, unsatisfactory—strong presence of foreign odours); texture (1—excellent, adequate gel texture, homogeneous, 2—very good gel texture with the presence of visible droplets of the oil phase, 3—good texture, slightly thin, with slight separation of the oil phase, 4—less good texture, thin with noticeable separation of the oil phase, 5—unsatisfactory texture, very thin, inhomogeneous with separating phases), spreadability (1—excellent typical for the product type, 2—very spreadable, slightly liquid, 3—well spreadable, moderately liquid, 4—poorly spreadable, liquid, 5—almost not spreadable, very liquid), absorbency (1—excellent and rapid absorption, leaves no sticky character, 2—very good absorption, no sticky film formation, 3—good absorption, leaves a slightly sticky impression, 4—poorer absorption with sticky film formation, 5—not absorbed, leaves a strongly sticky film). Spreadability and absorbency were evaluated during and after application of gels in the amount of 0.1 mL on an area of 8 cm^2^ of the volar side of the left forearm.

### 4.9. Statistical Analysis

Data from antimicrobial activity tests (disk and well diffusion method) were expressed as the mean ± standard deviation (SD). Statistical analysis was carried out by a one-way ANOVA followed by a Tukey test using Statistica software version 10, StatSoft, Inc. (Tulsa, OK, USA) at the significance level of *p* < 0.05.

The Kruskal–Wallis test was used to compare the sensory trait using scales. Ordinal tests were evaluated by Friedman’s test. The results of the sensory evaluation were processed at the 5% significance level (*p* < 0.05) using Unistat 5.5 software (Unistat Ltd., London, UK).

## 5. Conclusions

Alternative formulations for the commonly used antimicrobial component (ethanol) in gels intended for hand disinfection were investigated. The antimicrobial effect of the developed formulations was tested against selected physiologically significant Gram-positive and Gram-negative bacteria and yeasts.

The synergistic action of MA and selected EOs in the gel formulations significantly supported the high inhibitory effects in both in vitro and in vivo experiments against tested microorganisms and on hands, respectively. The prepared GEO/MA gel showed higher antimicrobial activity than the commercial ethanol-based samples. The stability test results confirmed the significant effect of temperature on gels containing only EO, while the addition of MA had rather a supporting and stabilising effect in the gel samples regarding their viscosity, unfortunately not with regard to antimicrobial efficacy. Sensory analysis has revealed that the organoleptic properties of GCEO/MA, GCLEO/MA and GLEO/MA were better than the commercial ethanol-based product.

It can be concluded that the cinnamon essential oil combined with mandelic acid provided an enhanced antimicrobial activity in comparison to formulations based only on EO, especially against yeasts. This combination of active compounds could serve as a natural alternative to the alcohol component. The prepared gel formulations containing essential oils (0.5 wt% EO) and mandelic acid (10–15% MA solution) can be applied as daily hand skin hygiene, mainly due to their extended efficacy and biocompatibility.

## Figures and Tables

**Table 1:** Inhibitory activity of mandelic acid (MA) solutions and four essential oils (EOs) on selected microbial strains using the disk diffusion method (inhibition zones include 6 mm paper disk).

	Inhibition Zone [mm]						
		*E. coli*	*P. aeruginosa*	*S. aureus*	*M. luteus*	*C. albicans*	*C. parapsilosis*
MA	5%	6.8 ± 0.6 ^a^	9.0 ± 0.6 ^b^	6.4 ± 0.5 ^a^	8.6 ± 0.5 ^a^	8.6 ± 0.5 ^a^	8.0 ± 0.5 ^a^
	10%	8.7 ± 0.5 ^b^	11.0 ± 0.7 ^c^	8.4 ± 0.6 ^b^	10.0 ± 0.6 ^b^	9.6 ± 0.6 ^b^	9.6 ± 0.7 ^b^
	15%	10.0 ± 0.7 ^c^	12.0 ± 0.6 ^d^	10.0 ±0.5 ^c^	16.0 ± 1.1 ^c^	10.0 ± 0.4 ^b^	10.0 ± 0.7 ^b^
EO	CEO	17.0 ± 0.5 ^d^	10.0 ± 0.5 ^c^	16.6 ± 0.4 ^d^	31.6 ± 1.9 ^d^	23.6 ± 1.0 ^c^	17.8 ± 1.2 ^c^
	CLEO	21.6 ± 0.6 ^e^	13.0 ± 0.8 ^d^	29.6 ± 0.9 ^e^	42.6 ± 1.3 ^e^	36.0 ± 1.3 ^d^	35.6 ± 2.4 ^d^
	LEO	- *^a^	- ^a^	10.6 ± 0.5 ^c^	11.6 ± 0.5 ^f^	15.0 ± 0.8 ^e^	21.0 ± 1.4 ^e^
	TEO	16.0 ± 0.6 ^f^	48.6 ± 1.6 ^e^	47.0 ± 2.1 ^f^	48.6 ± 1.5 ^g^	36.0 ± 0.9 ^d^	23.6 ±0.9 ^f^

^a–g^ Different lower case letters in the same column for each microorganism indicate significant differences (*p* < 0.05) (ANOVA); * no inhibition zone. CEO—cinnamon EO, CLEO—clove EO, LEO—lemon EO, and TEO—thyme EO.

**Table 2:** Inhibitory activity of gels containing essential oils (EOs) and/or mandelic acid (MA), or ethanol (Et) on selected microbial strains using the well diffusion method (inhibition zones include 6 mm well diameter).

		Inhibition Zone [mm]					
	*GMA*	*E. coli*	*P. aeruginosa*	*S. aureus*	*M. luteus*	*C. albicans*	*C. parapsilosis*
	5%	- *^a^	- ^a^	9.0 ± 0.8 ^b,c,e^	- ^a^	- ^a^	- ^a^
	10%	- ^a^	- ^a^	10.0 ± 0.6 ^c^	- ^a^	- ^a^	- ^a^
	15%	- ^a^	7.0 ± 0.5 ^b^	11.0 ± 0.6 ^d^	- ^a^	- ^a^	- ^a^
	***GEO***						
	CEO	11.0± 0.6 ^b^	9.0 ± 0.4 ^c^	11.6 ± 0.6 ^d^	- ^a^	9.0 ± 0.6 ^b^	9.0 ± 0.6 ^b^
	CLEO	7.6 ± 0.4 ^c^	9.6± 0.4 ^d^	10.0 ± 0.9 ^c,e^	- ^a^	14.1 ± 0.7 ^c^	17.0 ± 0.9 ^c^
	LEO	- ^a^	- ^a^	- ^a^	- ^a^	- ^a^	- ^a^
	TEO	13.6 ± 0.7 ^d^	8.0 ± 0.6 ^e^	10.6 ± 0.7 ^c,d^	12.0 ± 0.7 ^b^	11.0 ± 0.7 ^d^	14.0 ± 0.6 ^d^
	***GEO/MA***						
10% MA	CEO	17.6 ± 0.8 ^e^	9.6 ± 0.5 ^c,d^	11.6 ± 0.8 ^d^	11.0 ± 0.4 ^c^	36.0 ± 1.3 ^e^	32.9 ± 1.4 ^e^
	CLEO	11.6 ± 0.5 ^b,f^	8.0 ± 0.5 ^e^	9.0 ± 0.5 ^e^	13.6 ± 0.7 ^d^	19.0 ± 0.9 ^f^	31.0 ± 1.4 ^e,g^
	LEO	-^a^	- ^a^	- ^a^	- ^a^	- ^a^	11.0 ± 0.8 ^f^
	TEO	12.0 ± 0.7 ^f^	8.0 ± 0.5 ^e^	10.6 ± 0.7 ^c,d^	13.6 ± 0.5 ^d^	15.9 ± 0.7 ^c^	29.6 ± 1.4 ^g^
15% MA	CEO	13.6 ± 0.6 ^d^	9.0 ± 0.5 ^c^	10.0 ± 1.0 ^c,d,e^	13.0 ± 0.6 ^d^	36.1 ± 1.2 ^e^	37.6 ± 1.4 ^h^
	CLEO	9.0 ± 0.7 ^g^	- ^a^	10.0 ± 0.6 ^c^	12.6 ± 0.8 ^b,d^	16.0 ± 0.9 ^c^	20.0 ± 1.1 ^i^
	LEO	8.0 ± 0.7 ^c,g^	8.6 ± 0.5 ^c,e^	8.0 ± 0.6 ^b^	11.0 ± 0.8 ^b,c^	9.0 ± 0.5 ^b^	12.6 ± 1.0 ^f^
	TEO	12.0 ± 0.7 ^b,f^	- ^a^	11.6 ± 0.8 ^d^	13.6 ± 0.7 ^d^	20.6 ± 1.4 ^f^	29.1 ± 0.9 ^g^
	***GEt***						
	45%	- ^a^	9.0 ± 0.5 ^c^	- ^a^	7.0 ± 0.4 ^e^	8.0 ± 0.5 ^g^	8.0 ± 0.6 ^j^
	60%	15.0 ± 0.4 ^h^	10.0 ± 0.6 ^d^	11.0 ± 0.7 ^d^	8.0 ± 0.7 ^f^	12.0 ± 1.0 ^d^	11.0 ± 0.8 ^f^

^a–g^ Different lower case letters in the same column for each microorganism indicate significant differences (*p* < 0.05) (ANOVA); * no inhibition zone. GEO—gel with EO, GMA—gel with mandelic acid, GEO/MA—gel with combination of EO and MA, GEt—gel with ethanol, CEO—cinnamon EO, CLEO—clove EO, LEO—lemon EO, and TEO—thyme EO.

**Table 3:** Rank sums for the sensory properties of the scale and ranking test of hand gels.

Rank Sums (Kruskal–Wallis Test)	GCEO/MA	GCLEO/MA	GLEO/MA	GTEO/MA	SANYTOL
Appearance and colour	304.5	354.5	288.0	389.0	494.0
Fragrance	289.5	375.0	198.0	528.0	439.0
Texture	315.0	329.5	359.0	293.0	533.5
Spreadability	316.0	301.5	369.0	289.5	554.0
Absorbency	409.0	394.0	390.5	404.5	232.0
Ranking test (Friedman test)	27.0	33.0	25.0	46.0	49.0

**Table 4:** The formulations of prepared hand gels.

Gel Type	Carbomer [wt%]	Ethanol [wt%]	Essential Oil [wt%]	Mandelic Acid [wt%]
GEt	0.5	45/60	-	-
GEO	0.5	-	0.5	-
GMA	0.5	-	-	5.0
GEO/MA	0.5	-	0.5	5.0

## Data Availability

Data sharing not applicable.

## Supplementary Materials

The following supporting information can be downloaded at: https://www.mdpi.com/article/10.3390/ijms24043855/s1.
