# Supplementary material for: Non-Alcohol Hand Sanitiser Gels with Mandelic Acid and Essential Oils
Source: Int J Mol Sci. 2023 Feb 14;24(4):3855. doi: 10.3390/ijms24043855 (PMC9961504; doi:10.3390/ijms24043855)
Supplement: Supplementary file 1 [file ijms-24-03855-s001.zip › ijms-2197400-supplementary.pdf]

Supplementary material to

Non-Alcohol Hand Sanitiser Gels with Mandelic Acid and Essential Oils

Pavlína Egner[1], Jana Pavlačková[1], Jana Sedlaříková[1], Pavel Pleva[2], Pavel Mokrejš[3], Magda Janalíková[2]

[1]Department of Fat, Surfactant and Cosmetics Technology, Faculty of Technology, Tomas Bata University in Zlín, Vavrečkova 275, 760 01 Zlín, Czech Republic
[2]Department of Environmental Protection Engineering, Faculty of Technology, Tomas Bata University in Zlín, Vavrečkova 275, 760 01 Zlín, Czech Republic
[3] Department of Polymer Engineering, Faculty of Technology, Tomas Bata University in Zlín, Vavrečkova 275, 760 01 Zlín, Czech Republic

In this supplementary material, the following figures and tables are presented:

Figure S1. The pH and viscosity measured in time for GCLEO and GCLEO/MA (a), GLEO and GLEO/MA (b), GTEO and GTEO/MA (c) gels.

Table S1. Antimicrobial effect against Gram-negative bacteria (*E. coli*), Gram-positive bacteria (*S. aureus*) and yeasts (*C. albicans*) during GCLEO/MA, GLEO/MA, and GTEO/MA storage at three temperatures (22 ± 1 °C, 37 ± 1 °C, and 50 ± 1 °C).

Table S2. The chemical composition of used EOs (Nobilis Tilia, Czech Republic).

(a)

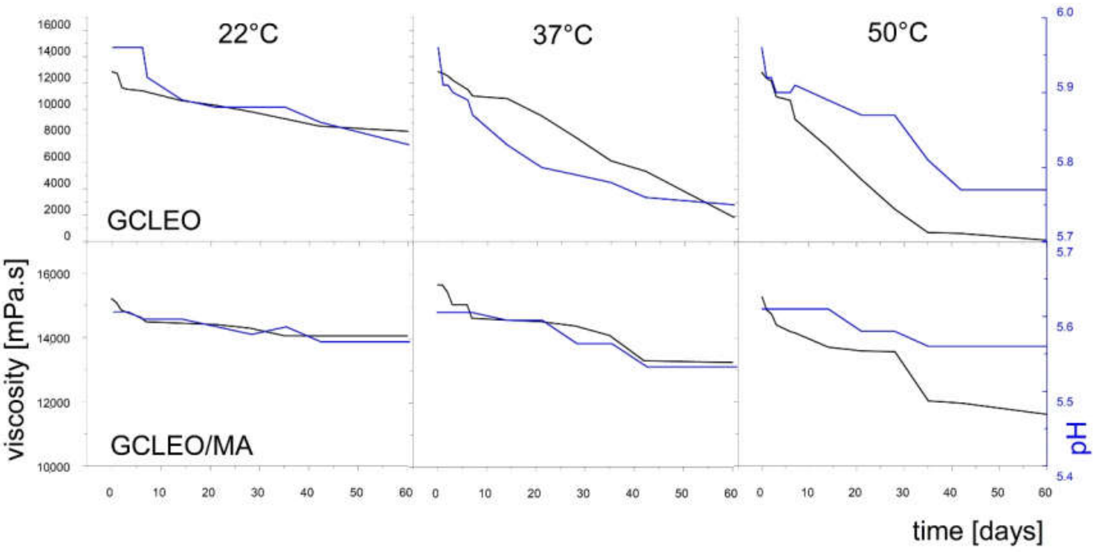

(b)

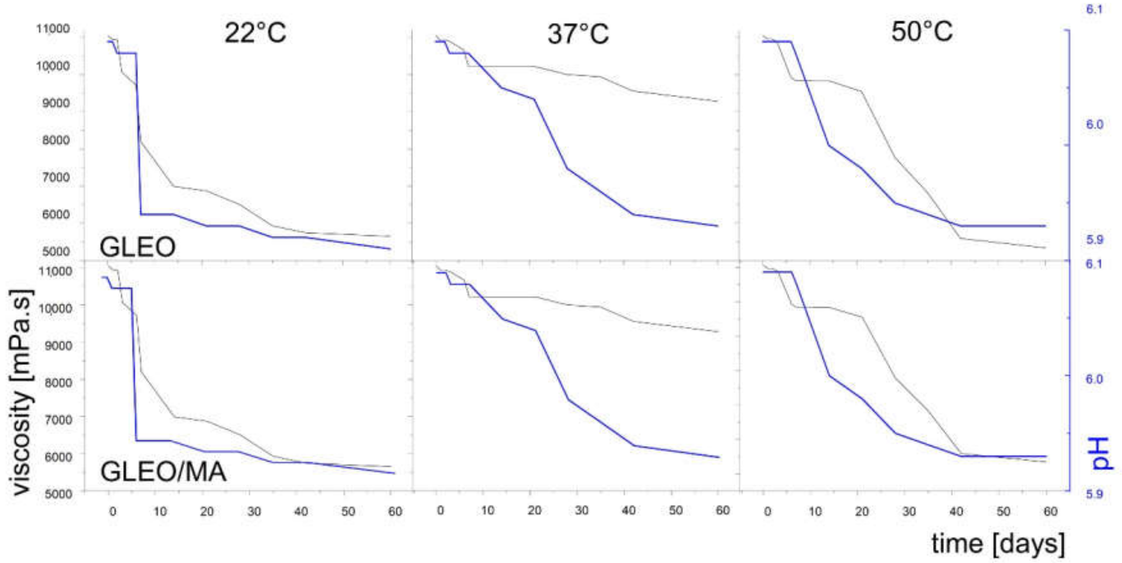

(c)

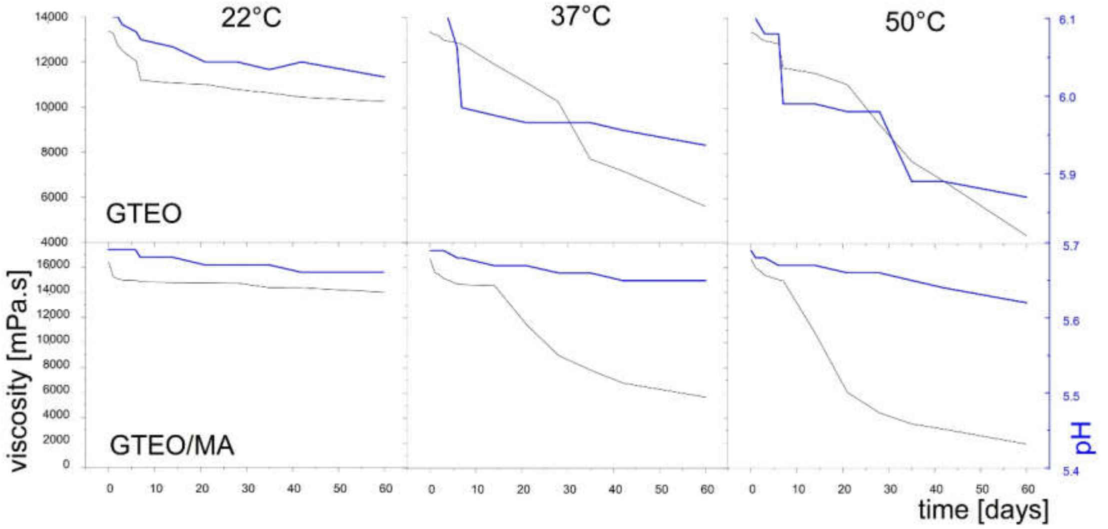

Figure S1. The pH values and viscosity measured in time for GCLEO and GCLEO+MA (a), GLEO and GLEO+MA (b), GTEO and GTEO+MA (c) gels.

Table S1. Antimicrobial activity (well diameter 6 mm) against Gram-negative bacteria (*E. coli*), Gram-positive bacteria (*S. aureus*) and yeasts (*C. albicans*) during GCLEO/MA, GLEO/MA, and GTEO/MA storage at three temperatures (22 ± 1 °C, 37 ± 1 °C, and 50 ± 1 °C).

		Inhibition zone [mm]								
		E. coli			*S. aureus*			*C. albicans*		
	°C /Day	0	28	60	0	28	60	0	28	60
GCLEO/MA	22	10	9	8	10	9	7	24	18	9
	37	10	8	6	10	6	6	24	9	6
	50	10	7	6	10	6	6	24	6	6
GTEO/MA	22	12	11	10	11	10	8	27	23	13
	37	12	9	7	11	9	7	27	14	6
	50	12	8	7	11	8	6	27	8	6
GLEO/MA	22	8	6	6	8	6	6	8	8	6
	37	8	6	6	8	6	6	8	6	6
	50	8	6	6	8	6	6	8	6	6

Table S2. The chemical composition of used EOs (Nobilis Tilia, Czech Republic).

**Component**	**Content in EO (%)**			
	**Cinnamon**	**Clove**	**Lemon**	**Thyme**
Coumarin	6.0–8.0	–	–	–
Limonene	< 9.0	–	54.0–74.0	–
Methoxycinnamaldehyde	< 5.0	–	–	–
(E)-Cinnamaldehyde	42.0–70.0	–	–	–
B-Caryophyllene	–	2.0–12.0	–	–
Eugenol	–	36.0–95.0	–	–
Eugenyl Acetate	–	11.0–22.0	–	–
β-Pinene	–	–	< 18.0	–
γ-Terpinene	–	–	2.9–14.0	1.0–5.0
Geranial	–	–	< 2.5	–
p-Cymene	–	–	0.3–1.1	20.0–30.0
Carvacrol	–	–	–	1.0–3.0
Linalool	–	–	–	1.0–5.0
Myrcen	–	–	–	1.0–5.0
Terpin-1-en-4-ol	–	–	–	1.0–5.0
Thymol	–	–	–	40.0–60.0
